# Biometric authentication security enhancement under quantum dot light-emitting diode display via fingerprint imaging and temperature sensing

**DOI:** 10.1038/s41598-023-28162-6

**Published:** 2023-01-16

**Authors:** Hanyung Jung, Soobin Sim, Hyunkoo Lee

**Affiliations:** 1 Department of Green Semiconductor Design Engineering, Korea Polytechnics, Seongnam‑si, Gyeonggi-do 13122 Republic of Korea; 2 Department of Electronics Engineering and Institute of Advanced Materials and Systems, Sookmyung Women’s University, Seoul, 04310 Republic of Korea

**Keywords:** Electrical and electronic engineering, Organic LEDs, Inorganic LEDs, Displays, Optical sensors, Optoelectronic devices and components, Graphene, Nanoparticles, Quantum dots, Graphene, Electronic devices, Sensors

## Abstract

We improved biometric authentication security using dual recognition based on fingerprint image detection and skin-temperature-change sensing under quantum dot light-emitting diode (QLED) displays. QLEDs are more advantageous than organic light-emitting diodes (OLEDs) in terms of the contrast classification of patterns such as those in fingerprint recognition, owing to their narrow full-width-half-maximum. In this work, scattered, transmitted, and reflected light was captured from the top of the QLED, improving the digital luminance by 25%, as compared with that of OLEDs, because the electroluminescence spectra of the QLED were sustained, whereas those of the OLED were distorted by the generated noise peaks. A QLED with eight apertures sized up to tens of micrometers, mimicking the actual wiring structure of commercialized smartphones, was implemented to detect human fingerprints. The QLED using reduced graphene oxide as the temperature sensor detected temperature changes instantaneously upon finger touch, showing a 2% temperature response based on the human body temperature; however, the temperature change was less than 0.1% for spoof fingerprints printed on paper. Thus, this study successfully enhanced biometric authentication security, through fingerprint recognition based on image sensing using an optical system with micrometer-sized apertures and skin-temperature detection under QLED displays.

Recently, financial transactions and online shopping using mobile devices have increased dramatically.^1–3^ The importance of biometric authentication in mobile devices has been increasing owing to its excellent security and convenience.^4–6^ Mass-produced smartphones such as Samsung Galaxy, released in 2020, use optical fingerprint recognition on screen.^7,8^ Financial transactions and shopping on smartphones require authentication technologies such as security numbers and fingerprints for the prevention of fingerprint spoofing and forgery.^9,10^ However, using only the authentication method increases the possibility of spoofing the financial system on smartphones.^11^ Addressing this issue, the dual biometric authentication of images and temperature sensing is expected to improve authentication security on smartphones.

Organic light-emitting diode (OLED) displays have been used as the main display panel in mobile smartphones owing to their excellent performance, including a wide color gamut, high contrast ratios, fast response times, and flexibility.^12–16^ However, the wide full-width-half-maximum (FWHM) of the electroluminescence (EL) spectra of OLEDs exhibits flaws as an appropriate light source for fingerprint recognition based on skin reflectance; this is because EL spectra with a wide FWHM can easily change when the light source interacts with human skin.^17^ Inorganic light-emitting diodes (LEDs) have an extremely narrow FWHM; however, it is difficult to fabricate self-emitting inorganic LED-based displays without color filters for mobile smartphones. As an alternative, quantum dot light-emitting diodes (QLEDs) offer the same merits as OLEDs and also possess an extremely narrow FWHM.^18–20^ Using a QLED light source, clearer fingerprint data can be obtained even after scattering from a finger, thereby increasing the accuracy of fingerprint sensing. However, thus far, only a few studies have investigated fingerprint recognition based on QLED light sources. Reduced graphene oxide (rGO) has received considerable attention owing to its high conductivity and solution processabilty.^21–23^ In addition, rGO has high sensitivity and fast response speed to temperature changes as shown in Table S1 in Supplementary information, and can be manufactured at a low cost compared with platinum, gold, and silver, which are widely used as typical temperature sensors.^24–26^ Accordingly, rGO can be an effective material for temperature sensor application.^27^

Herein, we report an improvement in biometric authentication using a QLED through fingerprint image detection and temperature-variation sensing. The light generated from a green QLED, after being scattered and reflected from the skin of fingers, was captured by a camera after passing through an optical system with eight apertures sized up to tens of micrometers; the captured image in case of a green QLED was clearer than that in the case of green OLED. Furthermore, an rGO temperature sensor was used to detect the temperature changes with finger touch. The resistance of rGO thin film was changed by a human finger temperature, and the rGO temperature sensor can distinguish an actual human fingerprint from a spoof fingerprint by detecting resistance variance. Thus, by simultaneously adopting fingerprint image detection and temperature-variation sensing, authentication security can be enhanced significantly.

## Results and discussion

### Temperature response

**Figure 1:**
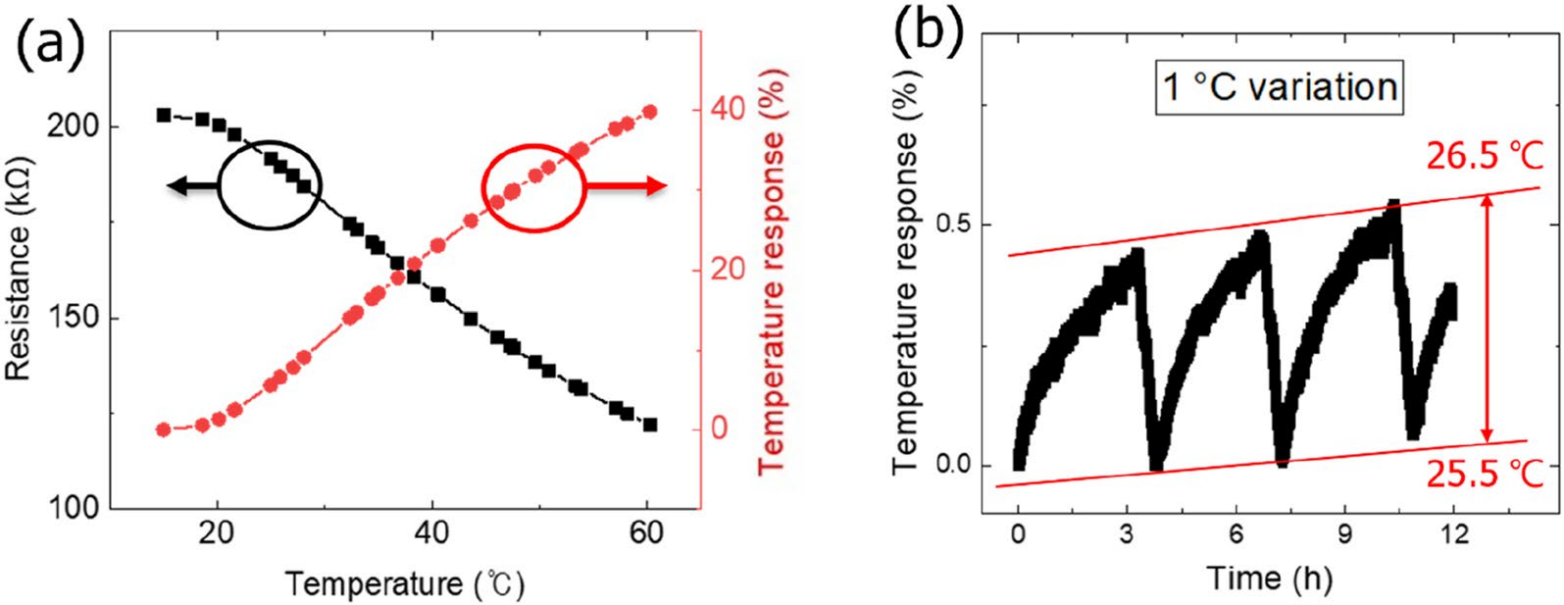
(**a**) Resistance variance and temperature response of rGO with respect to temperature sweep from 15 to 60 °C. (**b**) Temperature response of rGO for temperature variation of 1 °C and long-term stability.

The rGO resistance was measured as 205 kΩ and 123 kΩ at 15 °C and 60 °C, respectively, as shown in Fig. 1a. The temperature response was calculated as follows:

1 $$ Temperature\; response = \left| {\frac{{R_{i} - R}}{{R_{i} }}} \right| \times {1}00(\% ), $$

where *R*_*i*_ and *R* are the initial resistance and the resistance at a particular temperature, respectively.^22^ Based on (1), the produced rGO sensor showed a 40% temperature response with a variation in resistance. The temperature was changed periodically at a rate of 0.25 °C/h for 12 h in a temperature chamber, as shown in Fig. 1b. The resistance changes indicated a repeatable temperature change rate, even with a change of 1 °C; a stable temperature change rate within 0.5% was also observed.

### Apertures size effects for image sharpness

**Figure 2:**
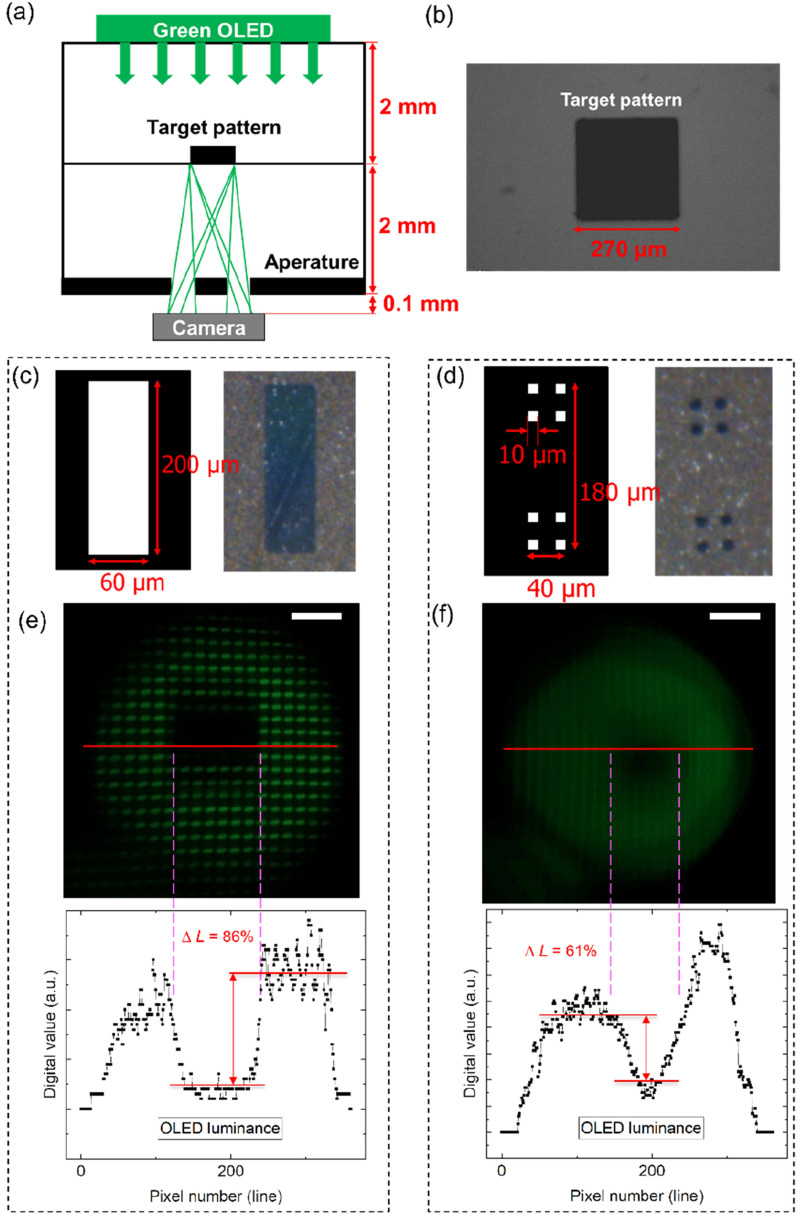
(**a**) Micro-sized aperture photomask structure with green light from a commercial OLED smartphone. (**b**) Actual target shape with a 270 µm square pattern. (**c**) 60 µm × 200 µm micro-sized aperture and actual pattern with optical microscope. (**d**) Eight 10 µm × 10 µm micro-sized apertures and actual pattern with optical microscope. (**e**) Captured image with 60 µm × 200 µm micro-sized aperture, scale bar: 200 µm and digital cross-sectional line luminance value of the captured image. (**f**) Captured image with a total of eight 10 µm × 10 µm apertures, scale bar: 200 µm and digital cross-sectional line luminance values.

Apertures were used for the pinhole effect to diminish the focal length because the gap between the display panel and camera was approximately 2 mm, as shown in Fig. 2a.^28^ A 270 µm square-shaped target fabricated on the photomask in Fig. 2b was passed through two types of apertures as shown in Fig. 2c,d: a 60 µm × 200 µm micro-sized aperture and eight 10 µm × 10 µm micro-sized apertures using the light (maximum green luminance: 381.5 cd/m^2^) of a commercial OLED smartphone. The obtained image files were directly converted into digital values using the Python programming language which was coded in house as shown in Fig. S1 in Supplementary information. The digital luminance value variance, *ΔL,* was calculated as follows:

2 $$ Luminance\;digital\;value\;variance = \left| {\frac{{L_{i} - L}}{{L_{i} }}} \right| \times {1}00(\% ), $$

where *L*_*i*_ and *L* are the initial luminance and digital luminance values at a specific position, respectively. The image obtained based on one large aperture was a sufficiently blur-free image showing each green sub-pixel of the commercial OLED; moreover, the pattern acquired by the center square also exhibited sharp edges, as shown in Fig. 2e. The target shape passing through the large aperture showed a luminance digital value variance of 86%, according to (2), whereas the target shape passing through several small apertures showed a luminance digital value variance of 61%, as depicted in Fig. 2e,f, respectively. However, in the case of several small apertures, although the green sub-pixels of the OLEDs were blurred and the total amount of light was insufficient, the square pattern at the center could be distinguished using the digital luminance value.

### Stack structure of touch sensor and pixel display panels for penetrated patterns

**Figure 3:**
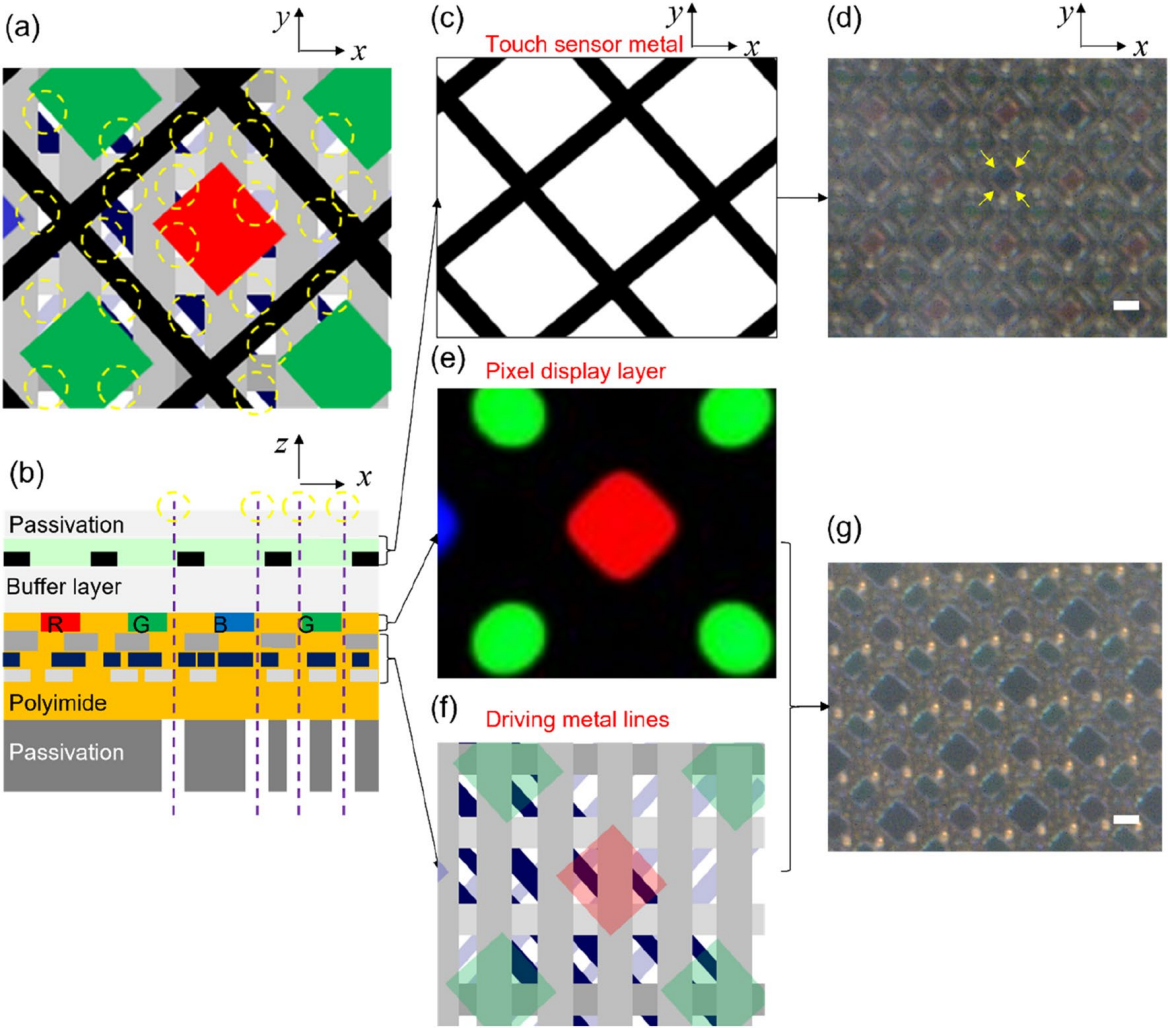
Schematic of stack structure for penetrated micro-sized holes patterns. (**a**) Combined layers with several penetrated micro-sized holes. (**b**) Cross-sectional view of the smartphone stack structure. (**c**) Schematic of touch metal sensor panel. (**d**) Optical microscope image of touch sensor metal lines aligned along the perimeter of sub-pixels (yellow arrow), scale bar: 20 µm. (**e**) Display layer with red, green, and blue sub-pixels. (**f**) Driving metal lines layer. (**g**) Optical microscope image of combined layers with pixel display layer and driving metal lines layer, scale bar: 20 µm.

In mass-produced smartphones, except for red, green, and blue pixels, the driving metal wiring and touch sensor panel wiring are densely formed. In typical OLED display panels in smartphones, the transparent portions of the white area within the yellow circle in Fig. 3a,b cover only a few dozens square micrometers per pixel. In the cross-sectional region of general smartphones, as shown in Fig. 3b, the transparent areas passing through several opaque layers of the upper and lower parts are formed arbitrarily. As shown in Fig. 3c,d, the uppermost layer is formed by surrounding the sub-pixel with a transmitter and receiver metal mesh pattern, which results in capacitance changes upon sensing touch. Under the touch sensor panel, the pixel display layer is patterned for the red, green, and blue pixels, as shown in Fig. 3e. Electric and driving wires of the electroluminescence pixel power supply (ELVDD), electroluminescence ground power supply (ELVSS), data, and transistor electrode lines for the emitting pixels are formed at the lower layer of the pixel display layer, as shown in Fig. 3f. Figure 3g shows an OLED display screen pixel image of a commercial smartphone. However, even the non-overlapping areas of commercial smartphone displays appear black under a microscope because of the passivation layer and the protective film that shields the display panel. Hence, to perform fingerprint sensing on a display screen, it is necessary to pattern or remove the opaque areas of the protective film under the display screen, as shown in Fig. 3b. Owing to the structure of commercial smartphones, only several transparent holes of 10 µm × 10 µm or less can be created in smartphone display panels. Therefore, employing multiple openings with a size of 10 µm is more advantageous for display panels in actual smartphone applications, as compared with using one large aperture.

### Different spectral properties of green OLEDs and QLEDs

**Figure 4:**
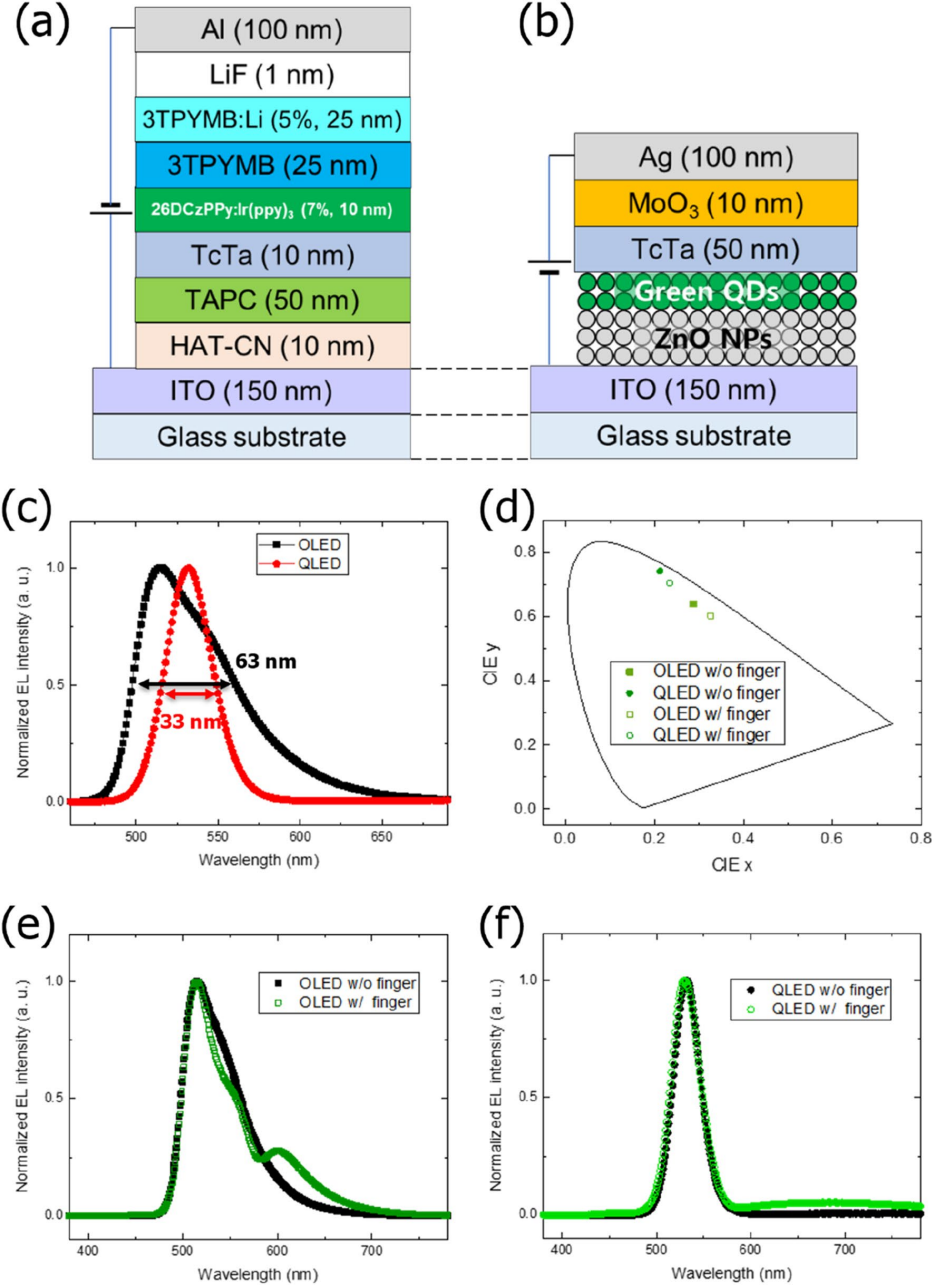
Device structures of (**a**) OLEDs and (**b**) QLEDs. (**c**) Normalized EL spectra of green OLED and QLED. (**d**) CIE 1931 color coordinates for EL spectra of OLED and QLED without (w/o) and with (w/) human finger. Normalized EL spectra of (**e**) OLED and (**f**) QLED w/o and w/ human finger.

Green OLEDs and QLEDs were fabricated as stack structures, as shown in Fig. 4a,b. The green OLED consisted of indium tin oxide (ITO) as the anode, 1,4,5,8,9,11-hexaazatriphenylene hexacarbonitrile (HAT-CN) as the hole-injection layer (HIL), 1,1-Bis((di-4-tolylamino)phenyl)cyclohexane (TAPC) as the hole-transport layer (HTL), 4,4′,4″-tri(N-carbazolyl)triphenylamine (TcTa) as the electron-blocking layer, 2,6-bis(3-(carbazol-9-yl)phenyl)pyridine (26DCzPPy) doped with tris(2-phenylpyridine)iridium(III) (Ir(ppy)_3_) as the green phosphorescent emitting layer (EML), tris(3-(3-pyridyl)mesityl)borane (3TPYMB) as the electron-transport layer (ETL), lithium (Li)-doped 3TPYMB and lithium fluoride (LiF) as the electron-injection layers, and aluminum (Al) as the cathode. The Green QLED consisted of ITO as the cathode, zinc oxide (ZnO) nanoparticle (NP) as the ETL, green quantum dots (QDs) as the green EML, TcTa as the HTL, MoO_3_ as the HIL, and silver (Ag) as the anode.

Figure 4c shows the normalized EL spectra of the fabricated green OLEDs and QLEDs at the same voltage of 6 V. The main emission peak and FWHM of the OLED and QLED are 515 nm, 63 nm, 532 nm, and 33 nm, respectively. The FWHM of the QLED is 30 nm narrower than that of the OLED. The Commission Internationale de l'Eclairage (CIE) 1931 color coordinates of OLEDs and QLEDs are (0.287, 0.640) and (0.212, 0.742), respectively, as shown in Fig. 4d. After the green light from the OLED and QLED was irradiated onto a human finger, the reflected light of the OLED and QLED EL spectra were measured to investigate the change in wavelength. The CIE 1931 color coordinates of the EL spectra of the OLED and QLED were (0.326, 0.603) and (0.234, 0.705), respectively. Thus, the changes in the CIE coordinates for OLED and QLED were 0.0537 and 0.0430, respectively, clearly indicating that change in the CIE coordinates for the OLED was 24.8% higher than that for the QLED when using the finger.

Although the main emission peak of the OLED was sustained, a shoulder peak at 554 nm and a long-wavelength peak at 599 nm appeared, as shown in Fig. 4e. However, the main EL spectra remained the same for the QLED after finger reflection, as shown in Fig. 4f. Although broad emissions between 600 and 780 nm appeared for the QLED due to light scattering,^29^ their intensities were extremely low. Light is absorbed by the various skin chromophores such as hemoglobin and melanin in the visible range and scattered because of the refractive index fluctuations on a microscopic level.^30^ The diffuse reflectance of light varies according to the amount of hemoglobin and melanin in the skin, the shape of human fingerprint and the amount of change in the refractive index of the tissue. In other words, after OLED or QLED light is reflected from a human finger, the spectrum can be changed as light of a specific wavelength is absorbed from hemoglobin and melanin, and some is scattered. For instance, oxyhemoglobin has high absorption at approximately 542 nm and 578 nm wavelength as shown in Fig. S2 in Supplementary information. Due to the strong absorption in this wavelength region, the light intensity at around 540 nm and 580 nm are reduced in the OLED spectrum, resulting in shoulder peak at 599 nm in Fig. 4e. In the case of the QLED, small overlapping area between EL spectrum of QLED and absorption spectrum of oxyhemoglobin owing to narrow FWHM of QLED compared with that of OLED resulted in relatively stable reflected EL spectrum. We calculated reflected spectra of OLED and QLED EL using human skin diffuse reflectance spectra.^31^ The reflected spectrum of OLED EL was dramatically changed depending on human skin with different melanin concentrations as shown in Fig. S3 in Supplementary information. On the other hand, the reflected spectrum of QLED EL was rarely changed regardless of human skin type. This result suggests that QLEDs have more stable reflected EL spectra than OLEDs and can, therefore, be useful as lighting sources for fingerprint recognition.

### Blurred degree of the pattern in OLED and QLED

**Figure 5:**
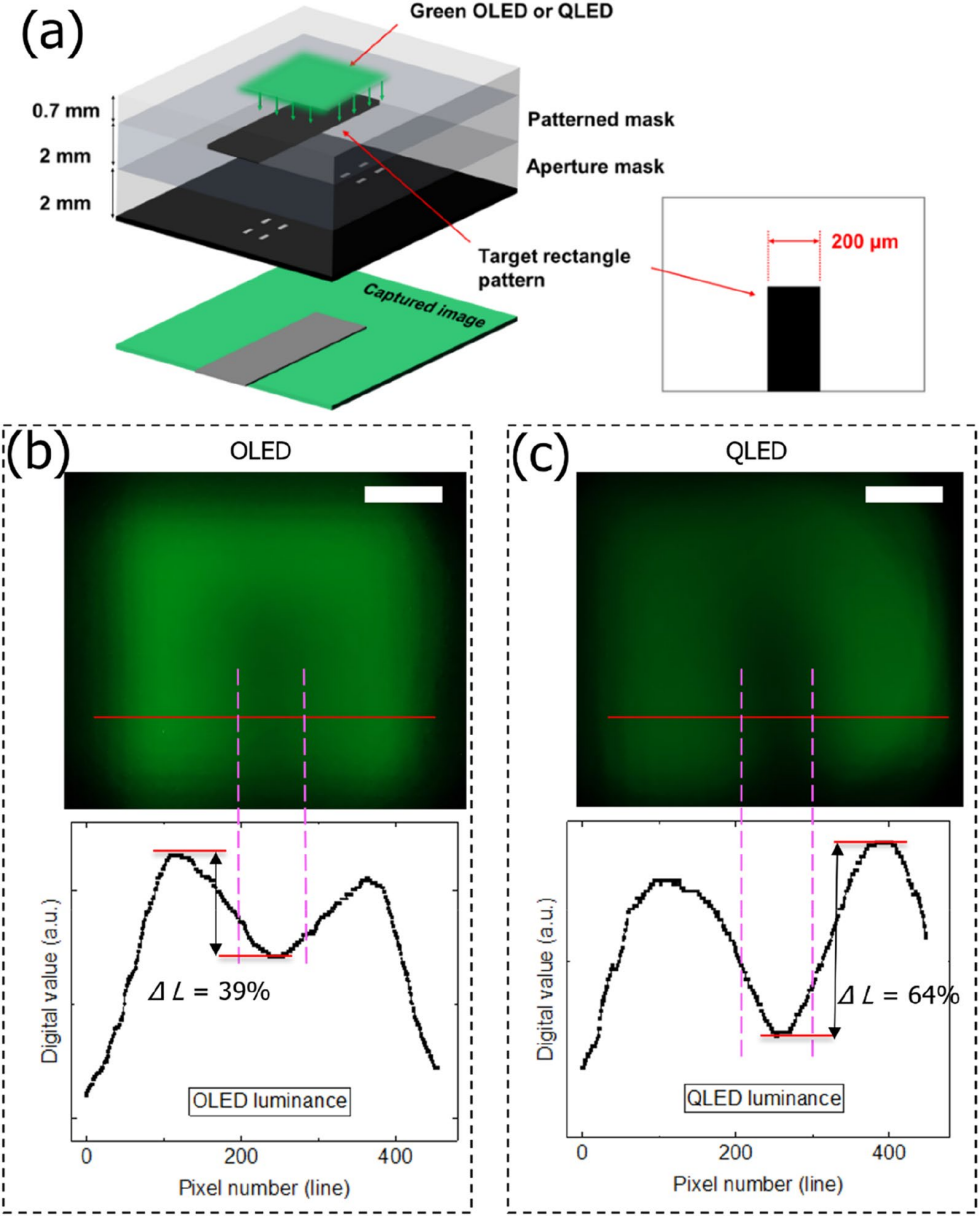
Blurred degree of the pattern in OLED and QLED green spectra. (**a**) Device structure with eight 10 µm × 10 µm apertures for direct illumination under OLED or QLED green light. (**b**) OLED and (**c**) QLED green light image under direct display pixel irradiation, scale bar: 200 µm. Cross-sectional digital line luminance values of the captured images of OLED and QLED green light.

To investigate the blurred degree of the pattern due to the difference in the characteristics of the green spectra between OLEDs and QLEDs, experiments were conducted to acquire a fine pattern through eight 10 µm × 10 µm micro-sized apertures in a stacked structure, as shown in Fig. 5a. Although the OLED and QLED exhibited different EL spectra, the same target pattern was used to capture the scattered OLED and QLED green light. In the photographed image, the contrast ratio of the QLED was larger than that of the OLED, as the rectangular pattern at the center was dark because light could not be transmitted, as shown in Fig. 5b,c. In addition, the digital image value of the acquired image of the QLED changed to 64%, according to (2), whereas the ratio of the minimum luminance to the maximum luminance of the OLED was 39%. Therefore, in the case of the QLED, the change in brightness was larger and less blurred occurred between the edges of the pattern, as compared with the case of the OLED.

### Fingerprint detection and temperature sensing

**Figure 6:**
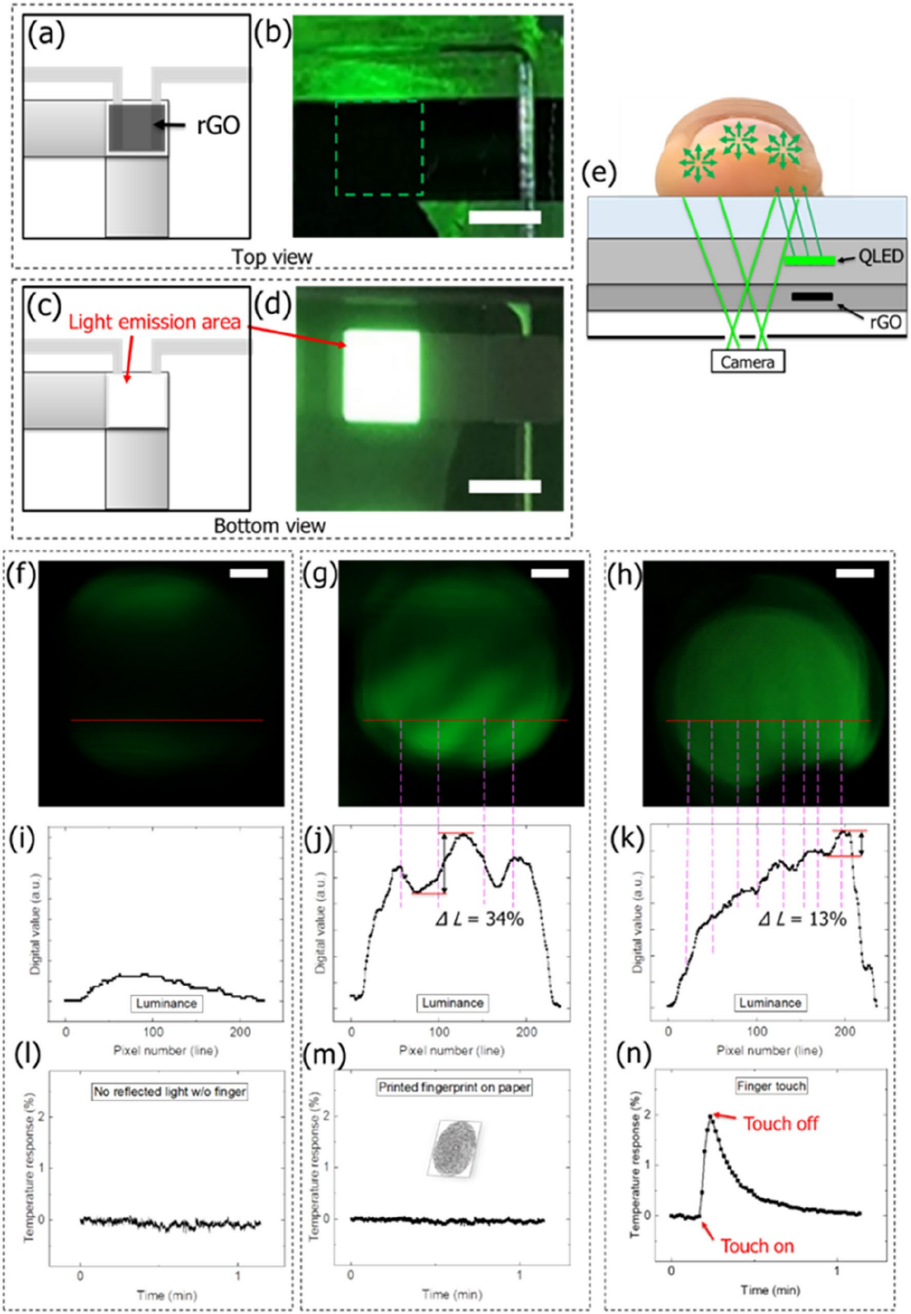
Fingerprint images and temperature-change obtained after passing light through micro-sized aperture. (**a**) Top view of QLEDs with rGO temperature sensor. (**b**) Top view of actual QLEDs, scale bar: 2 mm. (**c**) Bottom view of QLEDs. (**d**) Image of green-light-emitting bottom side of QLEDs, scale bar: 2 mm. (**e**) Device structure of QLEDs with eight 10 µm × 10 µm apertures and rGO temperature sensor for reflected and scattered light with human finger touch. (**f**) Captured image without fingerprint in dark chamber, scale bar: 200 µm. (**g**) Captured image of spoof fingerprint printed on paper with QLED green light, scale bar: 200 µm. (**h**) Captured image of human fingerprint with QLED green light, scale bar: 200 µm. (**i**) Cross-sectional digital green luminance values without fingerprint in dark chamber. (**j**) Cross-sectional digital green luminance values with spoof fingerprint printed on paper. (**k**) Cross-sectional digital green luminance values with human fingerprint. (**l**) Temperature response without fingerprint in dark chamber. (**m**) Temperature response with spoof fingerprint printed on paper. (**n**) Temperature response depending on human finger touch.

In general, there are various wiring lines that drive the OLEDs in commercial smart phones.^32–34^ Metal mesh lines with sizes of several micrometers are also present for the touch sensor panels aligned with the pixels.^35^ There was no direct light emission, as shown in Fig. 6a; only indirectly reflected light that penetrated the human finger skin was observed; it then scattered and re-emerged. Although there were already many areas that appeared dark in the top view of the QLEDs due to the desiccant, rGO was aligned to the rear of the pixel, where graphene oxide (GO) was patterned via guiding with Kapton® tape, as shown in Fig. 6b. Figure 6c shows an image of the light emission area viewed from the bottom and the image of the actual green-light-emitting QLED, as shown in Fig. 6d.

We investigated whether fingerprints could be obtained using a camera after passing through a micro-sized aperture between the combined wires, as shown in Fig. 6e. When a human finger was irradiated by the QLED green light, the light entering the finger skin was scattered, and a part of the light emerged along all directions due to scattering.^36^ Once the scattered light from the finger skin passed through the eight 10 µm × 10 µm apertures, images were acquired using a camera in a dark chamber, as shown in Fig. 6f. The captured images indicated a dark state because the finger was not raised on the bottom of the QLED light. Moreover, the shape of the fingerprint was determined based on spoof fingerprints printed on paper, as shown in Fig. 6g. The spoof fingerprints printed on paper showed high and low levels in terms of the digital image and could be distinguished. As shown in Fig. 6h, fingerprint ridges and valleys with a relatively low contrast ratio were obtained from the scattered light originating from the skin of the actual human fingerprints. Although the digital image contrast was low for the actual human fingerprints, luminance fluctuations were discerned between ridges and valleys. The image of fingerprint ridges and valleys were also obtained using the OLED light source, but its contrast ratio is lower compared with that of QLED as shown in Fig. S4 in Supplementary information. Consequentially, in case of the spoof fingerprints printed on paper, the obtained image was clearer than that in case of actual human fingerprints, because of the sharp contrast ratio difference.

As shown in Fig. 6i, the cross-sectional digital green luminance values of the captured image without a finger in the dark room reflected negligible QLED light; accordingly, the digital luminance values were relatively low. The cross-sectional digital green luminance values of the captured images with the spoof fingerprints printed on paper showed a digital luminance value variance of 34% between the highest and lowest values within adjacent ridges and valleys, according to (2), as shown in Fig. 6j. Meanwhile, the cross-sectional digital green luminance value obtained using the actual human fingerprint showed a digital luminance value variance of 13%, as shown in Fig. 6k, indicating the detection of a blurred fingerprint image.

Low temperature variations were observed in the dark room environment without reflected light, as shown in Fig. 6l. Similarly, the temperature response changed to less than 0.1% for the spoof fingerprints printed on paper, as shown in Fig. 6m. However, for the actual human finger touch, a 2% temperature response was obtained. When the temperature response was 0.5%, it took 15 ms as shown in Fig. S5 in Supplementary information. On removing the finger, the temperature response reverted to its original state within 1 min, as shown in Fig. 6n. In this manner, biometric authentication security was enhanced through combinational method of fingerprint and skin temperature sensing using the QLED display.

## Conclusion

We improved biometric authentication security through fingerprint image detection and temperature-change sensing, simultaneously. The green QLED light source showed improved fingerprint image detection compared with green OLED light source. An optical system on a QLED green light source was implemented comprising eight apertures sized up to several tens of micrometers for mimicking a practical smartphone display panel structure. On touching a finger on the QLED screen, the scattered, transmitted, and reflected light in the skin was captured using a camera on the bottom of the QLED and the digital luminance values of the obtained images were increased. Furthermore, the fabricated device detected temperature change and distinguished real human fingerprint from printed fingerprint on paper. Therefore, our device structure can be useful for enhancing biometric authentication security in the QLED-based mobile devices.

## Materials and methods

### Green OLEDs and QLEDs

ITO-patterned glass substrates were sequentially cleaned with acetone, methanol, and deionized water using ultrasonic cleaner, for green OLEDs and QLEDs fabrication. All the organic materials and top cathode metals were deposited in succession using the vacuum thermal evaporation method without breaking vacuum, onto the dried ITO-patterned glass substrates for OLEDs. During the deposition of the doping layers, the deposition rates of both the host and dopant materials were controlled simultaneously using a quartz crystal oscillator.

For QLEDs, ZnO NP thin films were deposited onto the dried ITO-patterned glass substrates via the spin-coating method, with 1.8% (weight per volume) ZnO NPs solution dispersed in 2-Propanol (isopropyl alcohol, IPA) at 2000 rpm for 30 s. After ZnO NP layer deposition, the processed films were dried for 20 min on a hot plate at 130 °C in air. Green QDs were subsequently deposited on the ZnO NP layers by spin-coating at 4000 rpm for 30 s, followed by drying for 1 h in a desiccator at a pressure below 10^–2^ Torr. Subsequently, the substrates were moved to a vacuum chamber, and organic, inorganic materials, and a metal were successively deposited by vacuum thermal evaporation at a pressure below 5 × 10^–7^ Torr. ZnO NPs and green QDs were purchased from infinityPV and ECOFLUX, respectively.

The fabricated OLEDs and QLEDs were transferred to a nitrogen-filled glove box, where they were encapsulated using UV-curable epoxy and a glass cap with a moisture absorbent. The emission area of the fabricated device was 2 mm × 2 mm.

The EL spectra were measured using a source-measure unit (Keithley-2450, Tektronics, U.S.A.) and spectroradiometer (CS-2000, Konica Minolta, Japan). The EL spectra of the OLEDs and QLEDs were measured at room temperature (approximately 293 K) in a dark box.

### Temperature sensor system

After the OLEDs and QLEDs were fabricated on a glass substrate, ITO electrodes were patterned as terminals with a 1 mm gap on the opposite side of the OLED and QLED light emitting direction, defined as the bottom side of the rGO temperature sensor. A 0.001 mL GO dispersion (0.6 mg/mL concentration) was drop-casted between the electrodes. The GO droplets were completely dried for photothermal reduction at 24 °C for 48 h. The GO sheets were reduced via the photothermal energy of laser irradiation (laser wavelength: 450 nm and power: 1 W).^37,38^ The edges of the ITO electrodes were glued with silver paste for decrease of contact resistance. The resistance across the rGO was measured by connecting ITO electrodes to multimeter (GDM-8351, GWINSTEK, Taiwan).

### Micro-size aperture systems

A pattern with 60 µm × 200 µm microscale apertures was fabricated using a photomask with a thickness of 2 µm. Similarly, a pattern with eight 10 µm × 10 µm micro-sized apertures was fabricated in a 40 µm × 180 µm area. After passing through the optical structure of the photomask, images were photographed using a smartphone camera (Xiaomi Redmi note 10, macro lens, shutter speed 1/4, ISO 100).

## Supplementary Information

Supplementary Information.

## Data Availability

The data that support the findings of this study are available from the corresponding author upon reasonable request.
