# Supplementary material for: Biometric authentication security enhancement under quantum dot light-emitting diode display via fingerprint imaging and temperature sensing
Source: Sci Rep. 2023 Jan 16;13:794. doi: 10.1038/s41598-023-28162-6 (PMC9842730; doi:10.1038/s41598-023-28162-6)
Supplement: Supplementary file 1 — Supplementary Information. [file 41598_2023_28162_MOESM1_ESM.pdf]

Supplementary information

Biometric authentication security enhancement under quantum dot light-emitting diode display via fingerprint imaging and temperature sensing

Hanyung Jung[1], Soobin Sim[2], and Hyunkoo Lee[2,*]

[1]Department of Green Semiconductor Design Engineering, Korea Polytechnics, Gyeonggi-do 13122, Republic of Korea; orcid.org/0000-0001-8362-7455

[2]Department of Electronics Engineering and Institute of Advanced Materials and Systems, Sookmyung Women's University, Seoul 04310, Republic of Korea.

Email: lhk108@sookmyung.ac.kr

Table S1. Temperature sensitive materials and performances.

Sensitive Material	Sensitivity (%/ ◦C)	Response Time
Reduced graphene oxide (rGO)	0.6345	1.2 s
Carbon nanotube	0.25	1~2 s
Ag	0.223	< 80 ms
Au	0.15	1.7–2.3 s
Ni fibers	0.48	-

```
import numpy as np
from PIL import Image
#import matplotlib.pyplot as plt
i=0
j=0
#img_name_oled = ('OLED.jpg')
#im_oled = Image.open('c:/'+img_name_oled)
img_name_qled = ('oled_square_direct.jpg')
im_qled = Image.open('e:/qdf/'+img_name_qled)
#pix_oled = np.array(im_oled)
#print(im_oled.size)
pix_qled = np.array(im_qled)
print(im_qled.size)

#while i<459:
#    print(pix_oled[i][100])
#    i = i + 1

print("\n\n pix_qled=====================")
while j<362:
    print(pix_qled[j][160])
    j = j + 1
```

Figure S1. Python code for obtaining digital values from the image file.

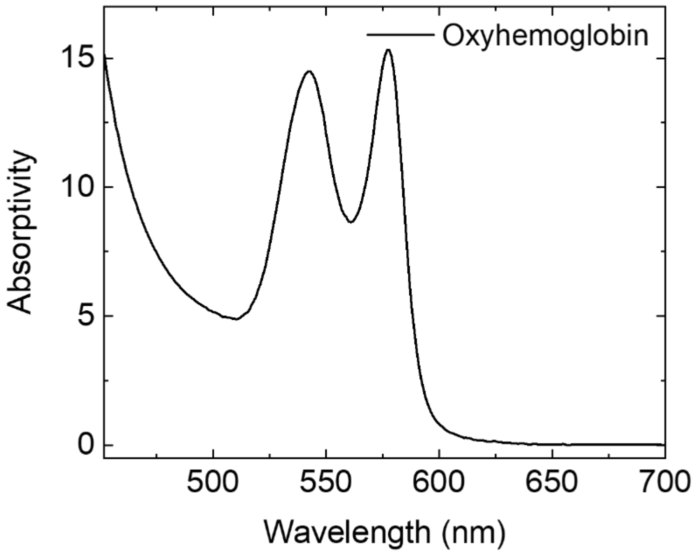

Figure S2. Absorption spectra of the common derivatives of human oxyhemoglobin in the visible range. The absorptivity is expressed in L·mmol^{-1}·cm^{-1}. (This data was obtained from the literature [S1])

[S1] W. G. Zijlstra and A. Buursma. Spectrophotometry of hemoglobin: absorption spectra of bovine oxyhemoglobin, deoxyhemoglobin, carboxyhemoglobin, and methemoglobin. *Comp. Biochem. Physiol.* **118B**, 743–749, (1997).

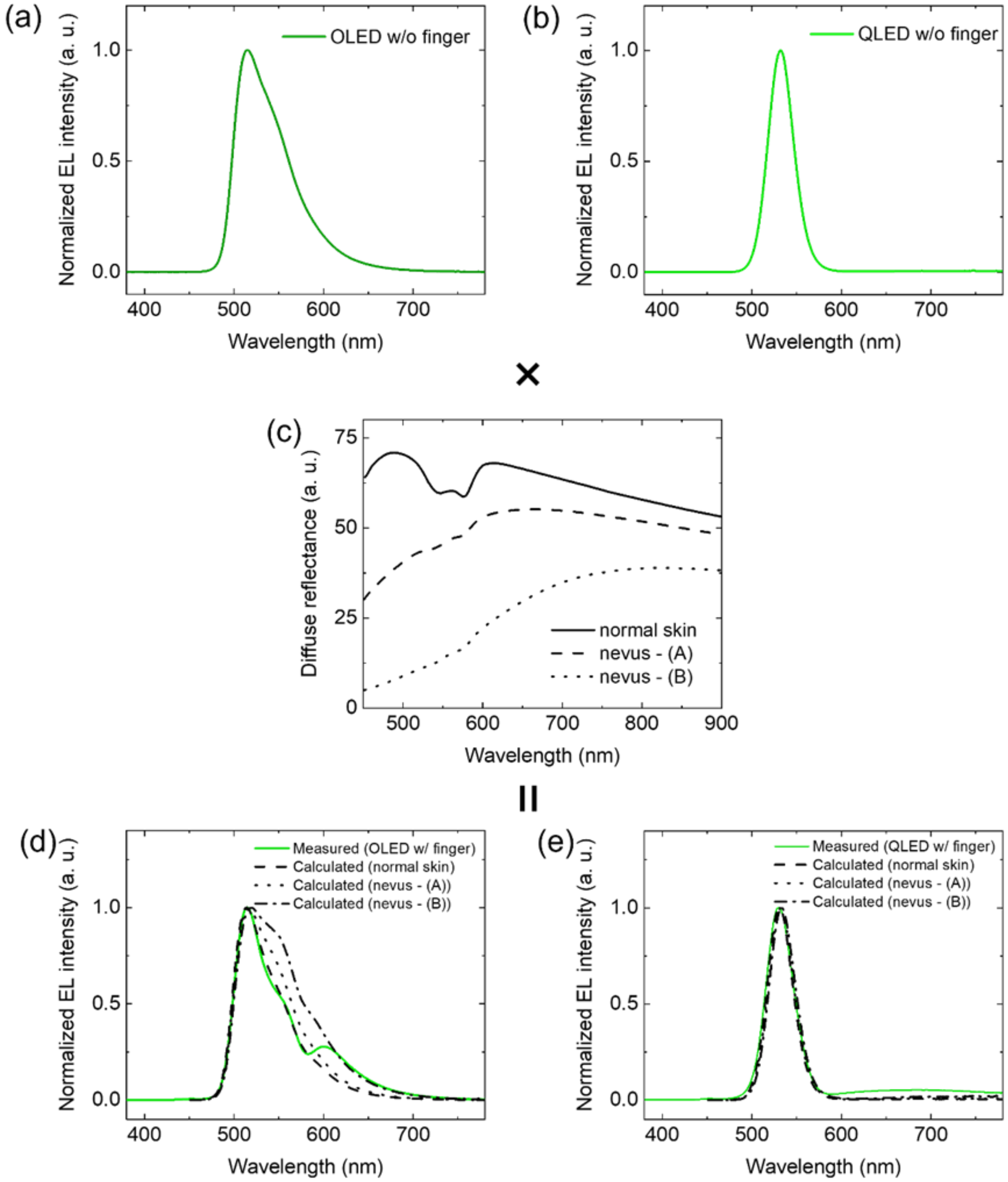

Figure S3. Normalized EL spectra of green (a) OLED, (b) QLED, (c) typical diffuse reflectance spectra from normal skin and from two different melanocytic nevi (This data was obtained from the literature [S2]) and measured and calculated reflected (d) OLED and (e) QLED EL spectra.

[S2] G. Zonios and A. Dimou Light scattering spectroscopy of human skin in vivo. *Opt. Express* **17**, 1256–1267, (2009).

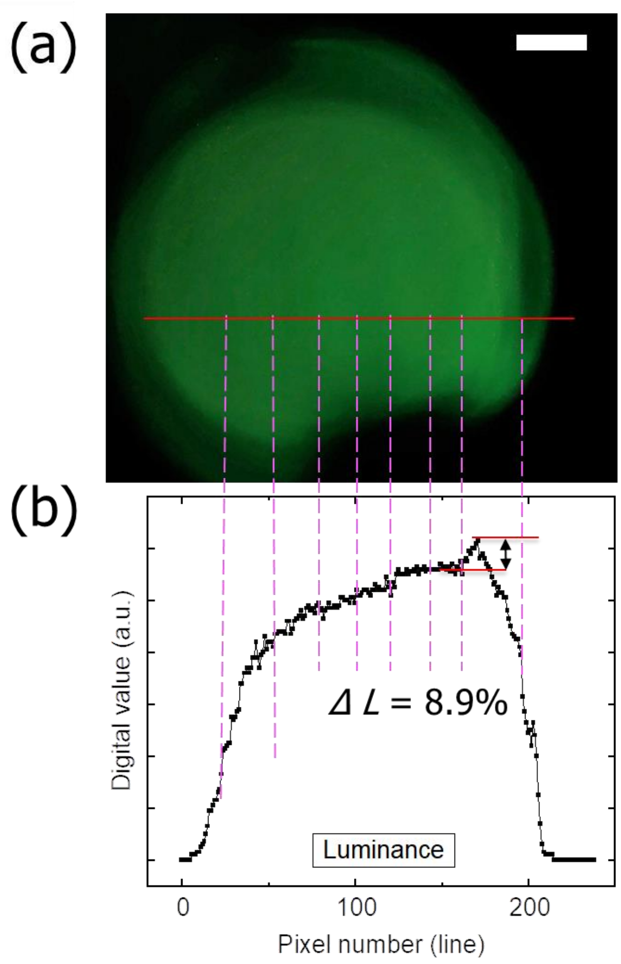

Figure S4. (a) Captured image of human fingerprint with OLED green light, scale bar: 200 μm and (b) cross-sectional digital green luminance values with human fingerprint.

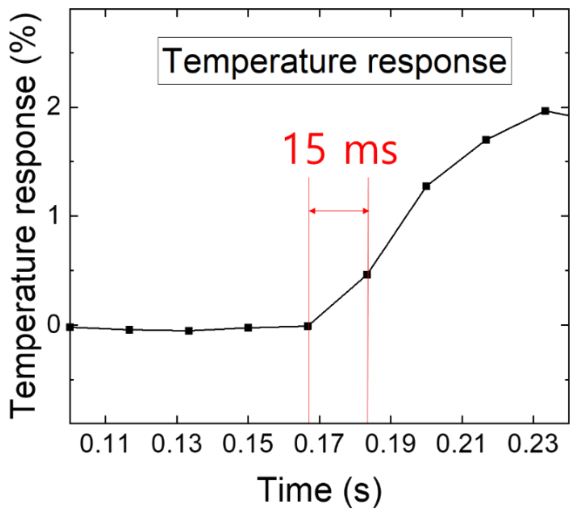

Figure S5. Rescaled graph of Figure 6(n) to magnify the moment in response to finger touches.
